# Bioactive Profiles, Antioxidant Activities, Nitrite Scavenging Capacities and Protective Effects on H_2_O_2_-Injured PC12 Cells of *Glycyrrhiza Glabra* L. Leaf and Root Extracts

**DOI:** 10.3390/molecules19079101

**Published:** 2014-06-30

**Authors:** Yi Dong, Mouming Zhao, Tiantian Zhao, Mengying Feng, Huiping Chen, Mingzhu Zhuang, Lianzhu Lin

**Affiliations:** 1 College of Light Industry and Food Sciences, South China University of Technology, Guangzhou 510640, China; 2 State Key Laboratory of Pulp and Paper Engineering, South China University of Technology, Guangzhou 510640, China

**Keywords:** *Glycyrrhiza glabra* L. leaf, nitrite scavenging capacity, tyrosinase inhibitory activity, PC12 cells, pinocembrin, liquiritin

## Abstract

This study compared the total flavonoid content of *Glycyrrhiza glabra* L. leaf and root extracts. Results suggested that the total flavonoid content in the leaf extract was obviously higher than that in the root extract. Pinocembrin, the main compound in the leaf extract after purification by column chromatography, showed good antioxidant activity and nitrite scavenging capacity, but moderate inhibitory effect on mushroom tyrosinase. Liquiritin was the main compound in root extract and possessed strong inhibitory effect on mushroom tyrosinase. Both compounds exhibited significant protection effect on H_2_O_2_-injured PC12 cells at a low concentration. These results indicate that *Glycyrrhiza glabra* L. leaf is potential as an important raw material for functional food.

## 1. Introduction

Licorice, which belongs to the legume family, is widely distributed in various areas of the world. Licorice distributed in Northwest China (including Xin Jiang, Inner-Mongolia, Gan Su and Ning Xia) performs the best quality [1]. There are three species used as licorice: *Glycyrrhiza* *glabra* L., *Glycyrrhiza uralensis* Fisch., and *Glycyrrhiza inflate* Bat. However, different specie of licorice possesses its own species-specific constituent. 

Licorice is one of the most ancient herb medicines worldwide. Previous researches proved that licorice possessed of therapeutic effects on peptic ulcers, skin infections, eczema, menopausal symptoms, inflammation, liver disease, respiratory ailments, chronic fatigue syndrome, Alzheimer disease, cancers, and even acquired immune deficiency syndrome (AIDS) [2]. Besides, licorice also has a long history of being applied as natural sweetener and flavor additive for preparing candies, chewing gum and beverage [3,4].

Licorice is always considered as the root and root-like stem in Traditional Chinese Medicine [2]. Basically, the aerial part of licorice was used as feed for cattle and flock, or burnt into fertilizer as the fuel. Previous studies focused on the root of licorice and pointed out that the root mainly contained triterpenes including glycyrrhetic acid and glycyrrhizin as well as flavonoids including liquiritigenin, liquiritin, isoliquiritigenin, isoliquiritin and coumarins [5]. However, there was little information about the chemical profile and biological activity of licorice leaf.

In order to make a full use of *Glycyrrhiza glabra* L., we comparatively evaluated the chemical profile (the total flavonoid content), antioxidant activity (oxygen radical absorbance capacity), inhibitory effect on mushroom tyrosinase and nitrite scavenging capacity of *Glycyrrhiza* *glabra* L. leaf and root extracts, separated and identified the main compound in the leaf extract as well. The protective effects of the main compounds in the leaf and root extracts on H_2_O_2_-injured PC12 cells were also evaluated. It is the first time to make a comparison of the chemical profiles and bioactivities between *Glycyrrhiza glabra* L. leaf and root extracts, which could be the theory guidance for making a full use of *Glycyrrhiza glabra* L.

## 2. Results and Discussion

### 2.1. The Total Flavonoid Content (TFC)

Flavonoids are some of the most important bioactive components of plants, especially in *Glycyrrhiza glabra* L. [5,6]. Therefore, TFC is an important indicator for evaluating the chemical profile of the leaf and root of *Glycyrrhiza glabra* L. The sodium borohydride/chloranil-based assay was chosen for measuring TFC in the leaf and root extracts. This assay can quantify almost all the flavonoids including flavonols, flavones, flavanols, flavononols, isoflavonoids, flavonones, and anthocyanins [7]. As shown in Table 1, TFC in leaf extract of *Glycyrrhiza glabra* L. was obviously higher than that in root extract. 

**Table 1:** The total flavonoid content, nitrite scavenging capacity, oxygen radical absorbance capacity and inhibitory effect on mushroom tyrosinase of tested samples.

	TFC (mg Catechin equiv/g)	ORAC Value (μmol trolox equiv/g)	Inhibitory Ability on Mushroom Tyrosinase (%)	Nitrite Scavenging Capacity (%)
Leaf	384.75 ± 4.11 ^a^	3339.26 ± 154.39 ^b^	28.66 ± 0.21 ^b^	63.24 ± 0.52 ^a^
Root	91.75 ± 6.61 ^b^	1812.91 ± 182.83 ^c^	45.32 ± 0.33 ^a^	49.19 ± 0.82 ^b^
pinocembrin	–	13904.28 ± 546.82 ^a^	10.14 ± 0.17 ^c^	67.51 ± 1.18 ^a^
Liquiritin	–	1121.01 ± 158.29 ^d^	23.13 ± 0.20 ^b^	5.87 ± 0.75 ^c^

^a–d^ means in the same column not sharing a common letter are significantly different (*p* < 0.01).

### 2.2. Oxygen Radical Absorbance Capacity

The oxygen radical antioxidant capacity (ORAC) assay is one of the most popular methods which based on hydrogen atom transfer (HAT) mechanism. It is the method that combines both the time and the degree of inhibition into the evaluation system, respectively [8]. ORAC is an assay for evaluating the antioxidant capacity of sample and is relevant to human. The ORAC value (shown in Table 1) was used for evaluating the antioxidant activity of different plant extracts including the licorice root extract [9,10]. The higher ORAC value means higher antioxidant capacity. In this study, the ORAC value of leaf extract was nearly two times higher than that of root extract. The ORAC value of the root extract was in agreement with the report of Kratchanova *et al.* [10]. However, there was rarely relative study on the ORAC value of *Glycyrrhiza glabra* L. leaf extract. Meanwhile, this result also showed that the extract with high TFC exhibited a high ORAC value, suggesting that the flavonoids might be one of the main active components for the antioxidant activities of *Glycyrrhiza glabra* L. leaf and root extracts. 

### 2.3. Nitrite Scavenging Capacity

Nitrite ions can induce some mutagenic and cell-damaging reactions in the acidic condition of the stomach. However, our daily diet makes us exposed to excess nitrite, which becomes a potential etiological factor for stomach and colorectal cancers. The nitrite scavenging capacity of samples increased by the decreasing of pH [11]. As shown in Table 1, the nitrite scavenging capacity of leaf extract was superior to that of root extract at pH 2.0. The modified method simplified the operation and was more close to the actual system in human body. It is the first time to evaluate the nitrite scavenging capacity of different part of *Glycyrrhiza glabra* L. comparatively. Additionally, the extract with high TFC exhibited strong nitrite scavenging capacity, which was also consistent with previous studies [12,13]. 

### 2.4. The inhibitory Effect on Tyrosinase

Tyrosinase is considered as the key enzyme in melanin biosynthesis. It contributes largely to form the color of mammalian hair and skin and prevents the skin from damaging by ultraviolet [14]. Previous investigations suggested that the chemicals with good tyrosinase inhibiting capacities could be used for evaluating the whitening effect of cosmetics and monitoring the color changes caused by some skin diseases [15]. Moreover, the reports also proved that flavonoids could inhibit mushroom tyrosinase. According to Fu *et al.* and Nerya *et al.* [5,14], the components including glabrene, isoliquiritigenin, licuraside, isoliquiritin, and licochalcone A in licorice root extract were proved to be tyrosinase inhibitors. However, there was rare research focused on the inhibitory effect of licorice leaf extract on tyrosinase. Research on the inhibitory effect on tyrosinase was meaningful to supply the theory for the application of sample for human health. In this study, *Glycyrrhiza glabra* L. leaf extract exhibited weaker tyrosinase inhibitory effect than that of root extract. This result suggested that the tyrosinase inhibitory capacity of sample might not be related to its TFC and antioxidant capacity. 

### 2.5. Identification of the Main Component in Glycyrrhiza Glabra L. Leaf

The main compound was obtained as white needle crystal in clusters. The mass spectrometry analysis indicated that the *m/z* of [2M-H]^−^ was 511.1. ^1^H-NMR (400 MHz, DMSO-*d*_6_,δ, ppm): 2.81 (dd, 1H, *J* = 17.1 Hz, 3-H), 3.16 (dd, 1H, *J* = 2.0 Hz, 2-H), 5.91 (d, 1H, *J* = 2.0 Hz, 6-H), 5.94 (d, 1H, *J* = 2.0 Hz, 8-H), 7.47–7.55 (m, 3H, 3',4',5'-H), 10.64 (s, 1H, 7-OH), 12.01 (s, 1H, 5-OH). The ESI-MS and 1H-NMR data were in agreement with the previous study [16,17]. Therefore, the compound was identified as pinocembrin.

### 2.6. HPLC Analysis

**Figure 1:**
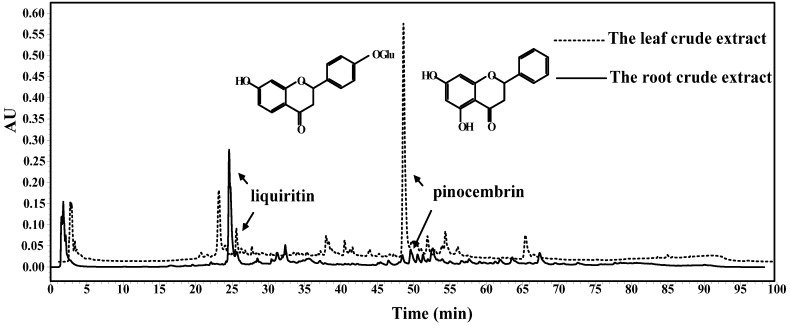
HPLC analysis of *Glycyrrhiza glabra* L. leaf and root extracts at the wavelength of 280 nm.

According to HPLC analysis of the *Glycyrrhiza glabra* L. leaf and root extracts in Figure 1, the components involved in leaf extract were different from those involved in root extract, especially the content and type of main component. 

As shown in Figure 1, liquiritin was widely distributed in the root extract of *Glycyrrhiza glabra* L., which was consistent with previous studies [4,18]. However, the content of liquiritin in leaf extract was inferior to that in root extract. Additionally, pinocembrin was determined as the main component in leaf extract. Nevertheless, the content of pinocembrin in root extract was relatively lower than that in leaf extract. According to the determination by HPLC, the content of liquiritin in the crude extracts of leaf and root were 18.21 ± 0.72 mg/g and 33.36 ± 0.56 mg/g, respectively. In addition, the content of pinocembrin was 58.90 ± 0.43 mg/g in leaf extract, while it was 6.51 ± 0.15 mg/g in root extract. Meanwhile, there were some other flavonoids and phenolics in the leaf and root extracts according to HPLC analysis, and the bioactivities of the leaf and root extracts were not only depending on the main compounds like pinocembrin and liquiritin, but also some other bioactive compounds like flavonoids phenolics and triterpenoids or the synergism of different bioactive compounds.

### 2.7. Bioactivities of Liquiritin and Pinocembrin

According to Table 1, the ORAC value of pinocembrin was more than 12 times higher than that of liquiritin, and the nitrite scavenging capacity of pinocembrin was superior to that of liquiritin, too. However, the inhibitory effect of liquiritin on mushroom tyrosinase was two times higher than that of pinocembrin at the same concentration (1 mg/mL).

As known, liquiritin and pinocembrin belong to the dihydroflavones. Liquiritin possesses two substituents, including the 7-OH on ring A and a 4'-*O*-glucose on ring B. However, pinocembrin owns two substituents including 5-OH and 7-OH on ring A. According to the previous studies, the *O*-dihydroxy structure in ring B and the 2,3-double bond could make a great contribution for radical scavenging activity [19]. However, the substitution on position 4' could bring a big blockade and decrease the radical scavenging activity of flavonoids [20]. The stronger hydrogen atom donation capacity of pinocembrin was due to two phenolic hydroxyl groups on ring A. However, 4'-*O*-glucose on ring B increased the steric hindrance, which decreased the hydrogen atom donation capacity of liquiritin. Meanwhile, due to the extra 5-OH on ring A of pinocembrin, the p-π conjugation was formed and the density of electron cloud was increased on ring A, the ring A and the 7-OH on ring A were activated both. The 4'-*O*-glucose on ring B of liquiritin also formed the p-π conjugation and increased the density of electron cloud on ring B, while caused little influence on ring A because of the distance. Based on the number of hydroxyl, the physical effect (steric hindrance) and the chemical effect (electron redistribution), pinocembrin suggested a better activity than liquiritin. The ORAC value relates to the hydrogen atom donation capacity of tested sample, which may be the reason for pinocembrin with a high ORAC value while liquiritin with a low ORAC value.

Pinocembrin (the main flavonoid in leaf extract of *Glycyrrhiza glabra* L.), exhibiting a higher oxygen radical antioxidant capacity and nitrite scavenging capacity than liquiritin (main flavonoids in root extract), contributed largely to the antioxidant and nitrite scavenging capacity of leaf extract. Although liquiritin possessed moderate antioxidant and nitrite scavenging capacity, it had relatively strong inhibitory effect on mushroom tyrosinase. Moreover, the inhibitory effect of root extract on mushroom tyrosinase was nearly two times stronger than that of leaf extract, implying that liquiritin contributed largely to the enzyme inhibitory effect of root extract. 

**Figure 2:**
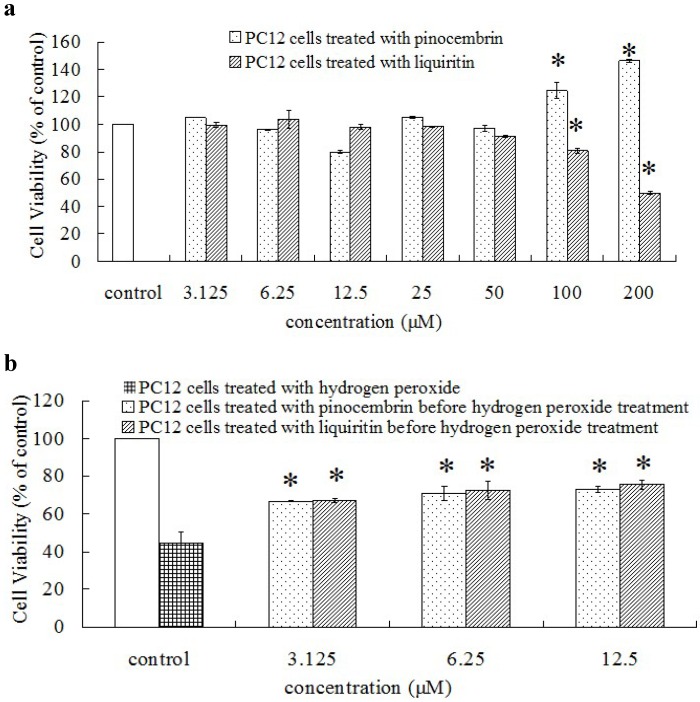
Protective effect on H_2_O_2_-injured PC 12 cells. □, negative control group without any treatment. (**a**) the cytotoxicity of the pinocembrin and liquiritin on cell viability in normal cells. * *p* < 0.01, significant differences between treatment groups and negative control group. (**b**) the protective effects of the pinocembrin and liquiritin on H_2_O_2_-induced oxidative damage on PC12 cells. * *p* < 0.01, significant differences between pretreatment with pinocembrin or liquiritin groups and H_2_O_2_-injured group without pretreatment.

As an important method to assess a potential health benefit of sample, rat adrenal pheo-chromocytoma cell (PC12 cells) was used for *in vitro* antioxidant model [21]. According to Gu *et al.* [21], a proper dose and treatment time (400 μM, 90 min) of H_2_O_2_ was chosen to obtain a proper cell model. As shown in Figure 2a, there were insignificant cytotoxicity of pinocembrin and liquiritin with low concentrations on PC12 cells (from 3.125 to 50 μM). However, the cell viabilities significantly decreased when treated with high concentrations of liquiritin and pinocembrin (100 and 200 μM) but increased when treated with high concentrations of pinocembrin (100 and 200 μM). Therefore, low concentration of pinocembrin and liquiritin were chosen for evaluating their protective effects on H_2_O_2_-induced oxidative damage on PC12 cells. 

According to Figure 2b, the cell viabilities of the treatment groups with pinocembrin and liquiritin were significantly higher than those of the negative control group. In addition, the pinocembrin and liquiritin exhibited the similar protection effects on H_2_O_2_-induced oxidative damage on PC12 cells at low concentrations (3.125, 6.25 and 12.5 μM). 

**Figure 3:**
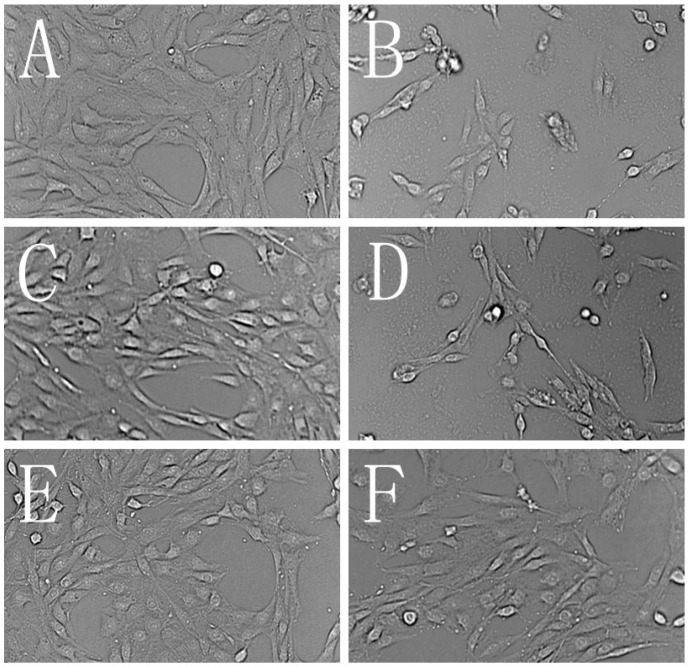
Morphological characteristics of PC12 cells. (**A**) Normal PC12 cells; (**B**) PC12 cells treated with 400 μM H_2_O_2_ for 90 min; (**C**) PC12 cells treated with pinocembrin at high concentration (200 μM) for 24 h; (**D**) PC12 cells treated with liquiritin at high concentration (200 μM) for 24 h; (E) PC12 cells treated with pinocembrin at 3.125 μM for 24 h before exposure to H_2_O_2_ (400 μM, 90 min); (**F**) PC12 cells treated with liquiritin at 3.125 μM for 24 h before exposure to H_2_O_2_ (400 μM, 90 min).

Figure 3 showed the morphological characteristics of PC12 cells under different treatments. PC12 cells were significantly damaged after being treated with 400 μM H_2_O_2_ for 90 min. As seen, cells were dispersed and the morphology was changed. The obvious cell shrinkage and membrane blebbing were observed. The protection effects of pinocembrin and liquiritin on H_2_O_2_-injured PC12 cells at 3.125 μM were observed according to Figure 3. 

As a typical chemical, H_2_O_2_ was considered as one kind of potential source for OH^−^ (one kind of the most dangerous radicals) and the most valuable exogenous ROS generator applied to investigation of oxidative stress and apoptosis [10,21]. Previous studies also indicated that flavonoids and phenolics could reduce the H_2_O_2_-induced oxidative damage on DNA and human cell (human vascular endothelial cell) [22,23]. In this study, the H_2_O_2_-induced cell damage of PC12 cells significantly decreased when treated with flavonoids (pinocembrin or liquiritin) at the proper concentration in cell culture (Figure 2b). 

## 3. Experimental Section

### 3.1. Materials

The plant materials used in this research were supplied by Xinjiang Tianshan Pharmaceutical Industry Limited Company (Xinjiang, China). The leaf and root were separated, sun-dried, pulverized with a laboratory mill (FW100, Taisite Instrument Co., Ltd., Tianjin, China) and stored in a glass desiccator at room temperature, respectively.

### 3.2. Chemicals

2,2'-Azobis (2-methylpropionamidine) dihydrochloride (AAPH), Trolox, fluorescein sodium salt, gallic acid, catechin, liquiritin, 3,4-dihydroxy-l-phenylalanine (l-DOPA), and mushroom tyrosinase were purchased from Sigma Chemical Co. (St. Louis, MO, USA). Acetonitrile of HPLC grade was obtained from Merck (Darmstadt, Germany). All other chemicals were of analytical grade. 

### 3.3. Preparation of Crude Extract

The leaf and root powder of *Glycyrrhiza glabra* L. (100 g) were extracted with 80% (v/v) ethanol with the solvent to material ratio of 20:1, in term of ultrasound-assisted extraction for 90 min, respectively. The extraction was performed in sonication water bath (KQ-800KDE, Kunshan Ultrasonic Equipment Co., Ltd., Jiangsu Kunshan, China) with the working frequency of 40 kHz and the power of 800 W at 60 °C. The supernatant was collected by centrifugation at 2,000 *g* for 10 min and the residue was extracted twice. The supernatants were combined, evaporated with a rotary evaporator (RE52AA, Yarong Equipment Co., Shanghai, China) under reduced pressure at 55 °C, and finally lyophilized by freeze-dryer (Marin Christ, Osterode, Germany) to obtain crude extract. The crude extract was stored in a refrigerator at −20 °C till further use. 

### 3.4. Extraction and Isolation of the Main Compound

**Figure 4:**
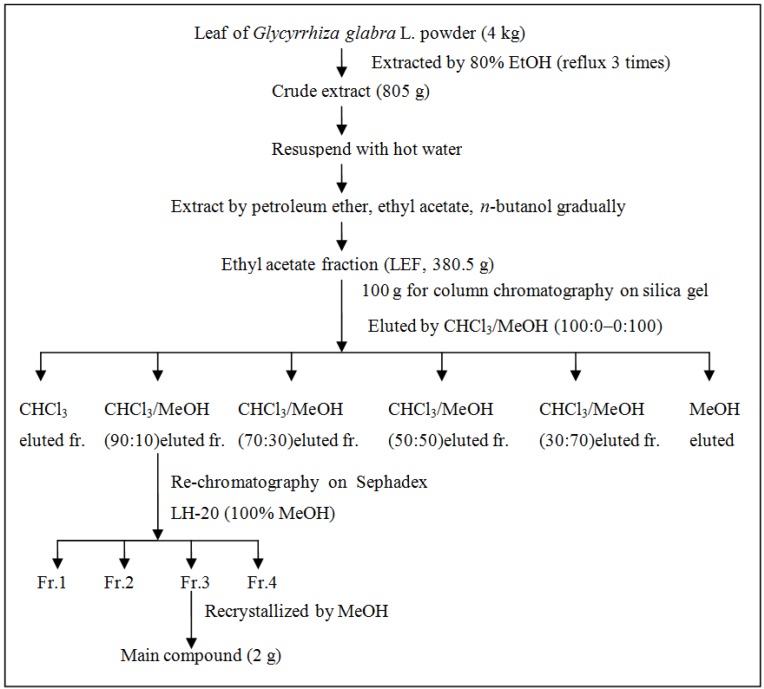
Isolation and purification of the main compound from ethyl acetate fraction (LEF) of *Glycyrrhiza glabra* L. leaf. Fraction 1, 2, 3 was abbreviated as Fr. 1, 2, 3.

Another batch of *Glycyrrhiza glabra* L. leaf (4 kg) were extracted with 80% (v/v) ethanol, in term of ultrasound-assisted extraction as illustrated above. The crude extract was extracted successively with 10 L of petroleum ether, ethyl acetate (EA) and *n*-butanol, respectively. The ethyl acetate fraction (LEF) was separated by silica gel (chloroform/methanol eluting ratio from 10:0 to 0:10) and Sephadex LH-20 (methanol eluting) to obtain the main compound. The steps for the extraction and isolation of the main compound were shown in Figure 4.

### 3.5. Molecular Weight Estimation and Identification

Assays for the molecular weight and NMR of sample were the same as our previous study [15].

### 3.6. High Performance Liquid Chromatography (HPLC) Analysis

HPLC analysis was performed using Waters 600 HPLC system (Waters, Milford, MA, USA). Waters (Milford, MA, USA) RP-C18 column (150 × 4.6 mm i.d., 5 μm) was chosen. Components of all samples were separated through gradient mobile phase consisting of distilled water (A) and acetonitrile (B). The gradient conditions were: 0–5 min: 5% B; 5–10 min: 5%–10% B; 10–30 min: 10%–30% B; 30–50 min: 30%–50% B; 50–70 min: 50%–60% B; 70–80 min: 60%–100% B; 80–85 min: 100% B; 85–90 min: 100%–50% B; 90–95 min: 50%–5% B; 95–100 min: 5% B. The solvent flow rate was 1 mL/min, the temperature was kept at 25 °C and the injection volume was 20 μL. According to the result of the full wave scanning of the crude extract, the detection wavelengths were set as 280 and 310 nm, respectively. 

### 3.7. Determination of Total Flavonoid Content (TFC)

A borohydride/chloranil-based assay method was used according to He *et al.* [7] with some modifications. An accurately weighed sample was dissolved with 1 mL of THF/EtOH (1:1 for v/v) in a graduated tube and then 1 mL of NaBH_4_ (50 mM) and 0.5 mL of AlCl_3_ (75 mM) were added. The tube was shaken (100 rpm) in thermostatic oscillator (THZ-82A, Changzhou Aohua Instrument Co., Ltd., Jiangsu, China) at room temperature for 30 min. Another 0.5 mL of NaBH_4_ (50 mM) was added into graduated tube and the tube was shaken for another 30 min at room temperature. Then, 2 mL of cold acetic acid (0.8 M, 4 °C) was added into each tube and the solution was mixed and kept in dark for 15 min. One milliliter of chloranil (20 mM) was added and the solution was kept in a thermostatic oscillator (100 rpm) at 95 °C for 60 min. The reaction liquid was cooled by tap water and made up to 4 mL with methanol. One milliliter of vanillin (1052 mM) and 2 mL of concentrated HCl (12 M) were gradually added into graduated tube. Then, the reaction mixture was kept in dark for 15 min. The absorbance of the reaction mixture was measured at 490 nm. The negative control group was consisted of all reagents and solvents except the sample. The total flavonoid content was determined using the catechin calibration curve, and expressed as milligrams of catechin equivalents per gram of dry mass of each sample. 

### 3.8. Assay of Oxygen Radical Absorbance Capacity (ORAC)

Assay of ORAC was determined according to our previous study [24].

### 3.9. Assay of Inhibitory Effects on Mushroom Tyrosinase

Inhibitory effects of samples on mushroom tyrosinase were determined according to our previous study [15]. 

### 3.10. Assay of Nitrite Scavenging Capacity

The assay of nitrite scavenging capacity were determined according to Liu *et al.* [25] with some modifications. Each sample (1 mL) was mixed with 1 mL of sodium nitrite solution (1 mM) and 5 mL of citrate buffer (0.2 M at pH 2.0). The reaction mixture was incubated at 37 °C in a water bath for 1 h. Then, 1 mL of reaction liquid was mixed with 5 mL of 2% acetic acid and 0.4 mL of Griess reagent (1% sulfanilic acid in 30% acetic acid and 1% naphthylamine in 30% acetic acid with the ratio of 1:1). The absorbance of the reaction mixture was measured at 520 nm after 15 min at room temperature, and the nitrite scavenging capacity (%) was calculated as follows:

Nitrite scavenging capacity (%) = (1 − (A_S_ − A_S0_)/A_0_) × 100% (1)

Where A_0_ is the absorbance of the negative control group without sample, A_S0_ is the absorbance of the sample solution and A_S_ is the absorbance of the treatment group with sample.

### 3.11. Assays of the Protection Effect on H_2_O_2_-Injured PC 12 Cell

#### 3.11.1. Cell Culture

PC12 cells line was purchased from the Cell Bank of Chinese Academy of Sciences (Shanghai, China). The cell was cultured in DMEM containing 100 U/mL of penicillin and streptomycin, 10% (v/v) fetal bovine serum in a fully humidified atmosphere in a 37 °C incubator containing 5% CO_2_. 

#### 3.11.2. The cytotoxic assessment

Assays of cytotoxic of the samples on PC12 cells were determined according to Gu *et al.* [21] with some modifications. The cell, with the content of 5 × 10^3^ cells/well, was seeded in a 96-well plate and incubated for 12 h. Then, the samples at different concentrations were added to treat the cells for another 24 h at 37 °C. The cell viability was determined using MTT method. 

#### 3.11.3. The Protection Effect of Flavonoids on H_2_O_2_-Injured Oxidative Damage

The assays of protective effects of samples on PC12 cells were based on the method from Gu *et al.* [21] with some modifications. Cell was seeded in 96-well plate with 5 × 10^3^ cells/well and incubated for 12 h. Then, samples at different concentrations were added to treat the cell and the cell was incubated for another 24 h. After that, 11 μL of H_2_O_2_ (400 μM) was added to treat the cell for another 90 min at 37 °C. The MTT method was used for determining the cell viability. The morphological inspection of the cell was detected by phase-contrast microscope (EVOS f1, AMG, Carlsbad, CA, USA).

### 3.12. Statistical Analysis

All tests were performed in triplicate, and the final experimental results were showed as mean ± standard deviation of three parallel measurements. Differences between variables were tested for significance by one-way ANOVA using SPSS 11 (SPSS Inc., Chicago, IL, USA). The mean values were considered significantly different when *p* < 0.01.

## 4. Conclusions

Based on the abundant flavonoids, phenolics and triterpenoids, the root of *Glycyrrhiza glabra* L. was widely used in the areas of medicine and food. In this study, all results suggested that the leaf of *Glycyrrhiza glabra* L. was rich in flavonoids and exhibited some good bioactivities (ORAC value and nitrite scavenging capacity). Pinocembrin, an important flavonoid in both propolis and honey [26,27], was also purified and identified as one of the main flavonoids in the leaf of *Glycyrrhiza glabra* L. Compared with liquiritin (one of the main flavonoids in the root of *Glycyrrhiza glabra* L.), pinocembrin also exhibited higher ORAC value, better nitrite scavenging capacity and significant protection effect at low concentration on H_2_O_2_-injured PC12 cells. All results stated above suggested that the extract of *Glycyrrhiza glabra* L. leaf could be used as an antioxidant or nitrite scavenger in food industry and pinocembrin separated from the leaf of *Glycyrrhiza glabra* L. could also used for functional food. Meanwhile, the utilization of the leaf could improve the whole economical value of *Glycyrrhiza glabra* L. 

The purification and identification of other flavonoids in the leaf of *Glycyrrhiza glabra* L. and their bioactivities will carry out in our future study. The fingerprint chromatography of flavonoids will be built based on the characteristic flavonoids obtained from the leaf of *Glycyrrhiza glabra* L., which come from different places at different seasons. Meanwhile, the extraction method and HPLC quantitation method (including the wavelength, LOD and LOQ) will be optimized.
